# *In vitro* antagonistic activity, plant growth promoting traits and phylogenetic affiliation of rhizobacteria associated with wild plants grown in arid soil

**DOI:** 10.3389/fmicb.2014.00651

**Published:** 2014-12-04

**Authors:** Wael S. El-Sayed, Abdellah Akhkha, Moustafa Y. El-Naggar, Medhat Elbadry

**Affiliations:** ^1^Biology Department, Faculty of Science, Taibah UniversityAlmadinah Almunawarah, Saudi Arabia; ^2^Microbiology Department, Faculty of Science, Ain Shams UniversityCairo, Egypt; ^3^Botany and Microbiology Department, Faculty of Science, Alexandria UniversityAlexandria, Egypt; ^4^Agricultural Microbiology Department, Faculty of Agriculture, Fayoum UniversityFayoum, Egypt

**Keywords:** arid soil, PGPR, 16SrRNA genes, ARDRA, phylogeny

## Abstract

The role of plant growth-promoting rhizobacteria (PGPR) in adaptation of plants in extreme environments is not yet completely understood. For this study native bacteria were isolated from rhizospeheric arid soils and evaluated for both growth-promoting abilities and antagonistic potential against phytopathogenic fungi and nematodes. The phylogentic affiliation of these representative isolates was also characterized. Rhizobacteria associated with 11 wild plant species from the arid soil of Almadinah Almunawarah, Kingdom of Saudi Arabia (KSA) were investigated. From a total of 531 isolates, only 66 bacterial isolates were selected based on their ability to inhibit *Fusarium oxysporum*, and *Sclerotinia sclerotiorum*. The selected isolates were screened *in vitro* for activities related to plant nutrition and plant growth regulation as well as for antifungal and nematicidal traits. Isolated bacteria were found to exhibit capabilities in fix atmospheric nitrogen, produce ammonia, indoleacetic acid (IAA), siderophores, solubilize phosphate and zinc, and showed an antagonistic potential against some phytopathogenic fungi and one nematode species (*Meloidogyne incognita*) to various extent. Isolates were ranked by their potential ability to function as PGPR. The 66 isolates were genotyped using amplified rDNA restriction analysis (ARDRA) and 16S rRNA gene sequence analysis. The taxonomic composition of the representative genotypes from both rhizosphere and rhizoplane comprised *Bacillus*, *Enterobacter* and *Pseudomonas*. Out of the 10 genotypes, three strains designated as PHP03, CCP05, and TAP02 might be regarded as novel strains based on their low similarity percentages and high bootstrap values. The present study clearly identified specific traits in the isolated rhizobacteria, which make them good candidates as PGPR and might contribute to plant adaption to arid environments. Application of such results in agricultural fields may improve and enhance plant growth in arid soils.

## Introduction

Soil, a dynamic, living matrix is an important resource for agricultural products. Soil is also a storehouse of microbial activity, which is confined to aggregates with accumulated organic matter, the rhizosphere. The rhizosphere both contacts plant roots and supports high populations of active microorganisms and it has attracted much interest (Nautiyal and DasGupta, 2007).

In the rhizospheric plant soil, diversity and community structure of microorganisms are plant species dependent and differ among varieties or cultivars. This may be affected by both specific plant root exudates and soil type (Kremer et al., 1990). Rhizospheric organisms can play a role in governing plant growth and development (Napoli et al., 2008).

Rhizobacteria that exert beneficial effects on plant development are termed “Plant Growth-Promoting Rhizobacteria” (Kloepper and Schroth, 1978). PGPR was found to be mainly involved in enhancing plant nutrition, stress tolerance or health (Vacheron et al., 2013). This is mainly due to their effect associated with enhanced availability of nutrients (Lugtenberg and Kamilova, 2009; Drogue et al., 2012), phytohormones-mediated stimulation of root system (Somers et al., 2004) and induced systemic resistance (Zamioudis and Pieterse, 2012).

PGPR include, for instance, Gram-positive PGPR taxa that include coryneform bacteria, *B. subtilis* and *B. cirulans*. Gram-negative PGPR includes fluorescent as well as non-fluorescent pseudomonads (*P. gladioli and P. cepacia*) and various members of the family Enterobacteriaceae. Also, nitrogen-fixing bacteria such as *Azospirillum, Herbaspirillum, Gluconacetobacter, Azotobacter* and *Azoarcus* have been reported as PGPR. These bacteria were mainly found to play a role in increasing nitrogen availability for plant nutrition and induction of minerals uptake (Bashan and de-Bashan, 2005).

Specific studies showed that PGPR either directly or indirectly promote plant growth and yield. The direct growth promoting mechanisms includes (i) N2 fixation; (ii) solubilization of mineral phosphate and zinc; (iii) sequestration of iron by production of siderophores; (iv) production of phytohormones such as auxins, cytokinins and gibberellins; (v) production of the enzyme 1-aminocyclopropane-1-carboxylate (ACC) deaminase, which hydrolyses ACC, the immediate precursor of ethylene in plants. Lowering of ethylene concentration in seedlings results in stimulating seedlings root length (Bashan and de-Bashan, 2005). PGPR also support plant growth indirectly, by improving growth restricting conditions via (i) production of antibiotics; (ii) depletion of iron from the rhizosphere; (ii) production of fungal cell wall lysing enzymes ß-(1,3)- glucanase and chitinase; (iii) synthesis of antifungal metabolites such as cyanide; (iv) competition for infection sites on roots; (v) induction of systemic resistance (Aeron et al., 2011; Gamalero and Glick, 2011; Jayaprakashvel and Mathivanan, 2011; Saraf et al., 2014).

Arid soils are dominant in the Kingdom of Saudi Arabia, but topographic differences and variations in soil composition suggest a significant number of bacterial species and plant associations may exist. A few literature reports suggest plant species surviving under such extreme conditions may harbor PGPR that have contributed to their fitness. Nonetheless, very little is known about the microbiota that colonizes the roots of desert plants (Jorquera et al., 2012).

The main objective of this study is to isolate rhizobacteria associated with some desert plant species and evaluate their potential contribution to the ability of such plants to survive under extreme conditions. The phylogenetic affiliation of the isolated PGPR and their antagonistic potential against phytopathogenic fungi and nematodes are also described.

## Materials and methods

### Site description

Almadinah Almunawarahis located at Eastern part of Alhijaz region in the Kingdom of Saudi Arabia at meeting-point Longitude (39°36′6″) and Latitude (24°28′6″). It is located in the north-western part of the kingdom, to the east of the Red Sea. The soils in the arid zone of Almadinah Almunawarah are coarse sandy textured and covered with sand dunes with low available water holding capacity, vulnerability to wind erosion and low fertility along with high salinity, calcareousness and gypsiferous nature. The annual average temperature during the year when the study was conducted was 30.2°C reaching 50°C in the summer months, annual total precipitation was 115 mm and annual average humidity was 17%, harsh conditions for most life forms.

### Plant species

A number of wild and native plant species collected during this study from different areas around Almadinah Almunawarah were identified according to the methodologies described by Chaudhary (1999, 2000, 2001). Identified plants included: *Zygophyllum simplex* L. (family *Zygophyllaceae*)*, Peganum harmala* L. (family *Zygophyllaceae), Notoceras bicorne* (Aiton) Amo (family *Brassicaceae*), *Cassia italica* (Mill.) (family *Caeslpinaceae*)*, Glinus lotoides* L. (family *Molluginaceae), Calotropis procera* (Ait.) Ait. (family *Asclepiadaceae*), *Tamarix amplexicaulis* Ehrenb. (family *Tamaricaceae*), *Capparis spinosa* L. (family *Capparidaceae*)*, Citrullus colocynthis* (L.) Schrad. (family *Cucurbitaceae*), *Tribulusterrestris* L. (family *Zygophyllaceae*) and *Haloxylon salicornicum* (Moq.) (family *Chenopodiaceae*).

### Fungal cultures and root-knot nematode

Cultures of two soil-borne pathogenic fungi namely *Fusarium oxysporum* (*F. oxysporum* f. sp. *lycopersici*), and *Sclerotinia sclerotiorum* were kindly provided by the Plant Pathology Department, Faculty of Agriculture, Ain Shams University, Egypt. The two fungal pathogens were maintained onto Potato Dextrose Agar (PDA) before use. A population of the root-knot nematode *Meloidogyne incognita* was routinely maintained on the susceptible tomato cultivar Castle Rock in the glasshouse at 27 ± 5°C in a box filled with sandy loam soil. Nematode eggs were extracted from heavily infested tomato roots using the extraction technique described by Hussey and Barker (1973). To promote the development of eggs and the hatching of second stage juveniles (J2), the nematode eggs/water suspension was kept in darkness at 24°C and aerated with an aquarium pump.

### Sample collection and isolation of rhizobacteria

Samples of rhizospheric soil and root system from 11 healthy wild plants were collected from different sites at Almadinah Almunawarah during the winter of 2011–2012. To estimate the number of root-associated rhizobacteria, 50 g of roots- adhered soils were used. From the selected plants, roots of three young and healthy plants were collected, shaken vigorously to remove loose soil, placed in sterile paper bags, and maintained in an ice-box. Six roots belonging to one plant species were pooled for an average sample. Sufficient portion of roots were aseptically placed in sterilized 250 ml conical flasks, and a solution of Tween phosphate buffered saline was added to give 100 ml final volume. The flasks were shaken, and tenfold serial dilutions were made in sterile 0.1 M MgSO4 (pH 7.0), and 0.1 ml aliquots from appropriate dilutions were spread- plated onto Tryptic Soy Agar (TSA) medium for total count. Each value presented is an average of three individual counts. The same procedure was followed to enumerate soil rhizospheric bacteria using soil surrounding roots. To estimate spore-forming bacteria from root segment and soil, 9 ml of each serial dilution was placed in a water bath at 80°C for 10 min to kill non-spore forming mesophilic bacteria. The heat-treated samples were serially diluted, inoculated onto TSA medium plates in triplicate and incubated aerobically at 30°C for 48 h (Bai et al., 2002). Developed colonies were counted and those representing different morphological types were selected, and further purified on TSA medium plates.

### *In vitro* screening for antagonism

Bacterial isolates were screened *in vitro* for growth inhibition of the worldwide distributed soil-borne phytopathogenic fungi *F. oxysporum*, and *S. sclerotiorum*. Briefly, 5 μl drops of each bacterial culture (10^8^CFUml^−1^) were streaked equidistantly on the margins of PDA plates adjusted to pH 7. Mycelial agar plug of 5 mm diameter from a 7-day-old culture of *F. oxysporum* or *S. sclerotiorum* grown on PDA plate was placed in the center of the plate between the two parallel streaks of the test bacterium. Control plates not inoculated with bacteria were also prepared. Two independent experiments with each bacterial isolate were performed. Plates were incubated at 25°C for 5 day. Antagonistic activity was assessed by relating mycelia diameter on plates inoculated with bacteria to mycelia diameter on control plates and computing percentage of Growth Inhibition (GI%).

To study the effect of bacterial filtrates on the egg hatching and mortality of *M. incognita*, three egg masses containing 300 ± 50 eggs per egg mass were mixed with 1 ml of the bacterial suspension in 1.5 ml Eppendorf tube and incubated at 28°C for 5 days. Equal numbers of egg masses in sterile TSA medium were kept as a control. The numbers of dead second-stage juveniles (J2) were recorded after 3 and 5 days using a light microscope and Hawksley counting slide.

### Screening isolates with PGPR traits on plant nutrition and growth stimulation

N2-fixation was tested by inoculating bacterial isolates and *E. coli* (as control bacteria) on plates of N-free agar medium (Haahtela et al., 1983) for 48 h at 28°C. The isolates that grow after being sequentially transferred 10 times to the same medium were considered as presumptive positive for N2-fixation. All isolates were tested for the production of ammonia as described by Cappuccino and Sherman (1992). Phosphate and zinc solubilization was tested by the dissolution of precipitated tricalcium phosphate [Ca3 (PO4)2] and zinc oxide (ZnO) respectively in agar medium (Saravanan et al., 2003; Rodriguez et al., 2004). The solubilization Index (SI) was calculated as the ratio of the total diameter (colony, halo zone) to the colony diameter (Edi-Premona et al., 1996). IAA production was tested according to the procedure described by Loper and Schroth (1986) using TSA medium supplemented with L-tryptophane.

### Antifungal and nematicidal traits

Siderophores production was detected as described by Pallai (2005). The assay used the ternary complex chrome azurol-S/Fe(III)/hexadecyl-trimethylammonium bromide as an indicator. Screening of bacterial isolates for hydrogen cyanide (HCN) production was done using cultures grown on TSA supplemented with glycine and alkaline picric acid as indicator (Castric, 1975). Chitinase production was assessed qualitatively by a microbiological method based on spotting of isolates on chitinase medium amended with colloidal chitin (Frändberg and Schnürer, 1998). Cellulase production was visualized by flooding the cellulose decomposition medium plates previously inoculated and incubated at 30°C for 8 days with 0.1% (w/v) Congo red for 15–30 min followed by bleaching the plates with 1M NaCl (Andro et al., 1984). Protease production was indicated by casein degradation after at least 4 days of incubation at 28°C (Abo-Aba et al., 2006). Salicylic acid (SA) production was assessed by the method of Leeman et al. (1996) using succinate medium.

### PCR amplification of 16S rRNA genes

A loopful from a bacterial colony was suspended in 50 μl of TE buffer (100 mM Tris HCl, 10 mM EDTA, pH 8.0), heated in a boiling water bath for 5 min, followed by cooling prior to PCR. One μl of cell extract was used as a PCR template to amplify the full length 16S rRNA gene (Arturo et al., 1995). An approximately 1500-bp fragment of the 16S rRNA gene corresponding to positions 8 and 1509 of the *E. coli* was amplified using the universal primer pair (27F: 5′-AGA GTT TGA TC[A/C] TGG CTC AG-3′, 1492R: 5′-G[C/T]T ACC TTG TTA CGA CTT-3′) (Lane, 1991). Amplifications were performed in a 50 μl reaction volume containing: 5 μl of 10× *Taq* buffer (100 mM Tris- HCl, pH 8), 1.25 mM MgCl2, 200 μM dNTPs (Invitrogen, USA), 1.2 μM forward primer and reverse primer set (Invitrogen, USA), 1U *Taq* DNA polymerase (Invitrogen, USA), and about 5 ng of template DNA.

PCR was performed in Thermal Cycler (Applied Biosystem 2720, USA). The PCR conditions were adjusted to 5 min for initial denaturation at 94°C and then 30 cycles of 1 min at 94°C, 1 min at 54°C, and 1 min at 72°C, and finally 10 min at 72°C. The amplified genes were subjected to electrophoresis using 1% agarose gel with the size markers (1 kb DNA ladder, Invitrogen, USA).

### Amplified ribosomal DNA restriction analysis (ARDRA)

Twenty microliters of each 16S rRNA gene amplicon was digested for 3 h at 37°C with 2.5 U *Hae*III (Fermentas, Vilnius, Lithuania). The DNA restriction fragments were subjected to electrophoresis using 3% agarose gel containing ethidium bromide (50 ng/ml). The gels were made visible by UV transillumination and digitized with the gel documentation system (Gel Doc XR System, Biorad, USA). The images for electrophoretic pattern were analyzed with GelCompar II software (Applied Maths, Kortjik, Belgium). The patterns were used to construct a dendrogram using the unweighted pair group method of arithmetic average (UPGMA) clustering algorithm and Dice similarity coefficient index. Bacterial isolates showing same pattern were grouped into the same group.

### Sequencing and analysis of 16S rRNA genes

The nucleotide sequence analysis of the selected isolates based on ARDRA profiles were determined by automated florescent dye terminator sequencing method (Sanger et al., 1977) using DYEynamic ET Terminator Cycle Sequencing Kit, Amersham Pharmacia Biotech with a model ABI 310 genetic sequence analyzer (Applied Biosystems, CA, USA) according to the user manual. One representative isolate of each genotypic profile was chosen for 16S rDNA partial sequencing. Dye terminator-based sequencing was performed using PCR-amplified segments of about 517 bases covering up V3 region of 16S rRNA genes using primer set of EUB8F/EUB517R. Amplified DNA was purified by ethanol precipitation to remove unincorporated dye-labeled terminators. The pellet was then dissolved in 20 μl formamide loading dye and heat shocked at 95°C for 2 min before injection to DNA sequence analyzer.

The sequences were analyzed by *Genetyx-Win* MFC application software version 4.0. Related sequences were identified using BLAST search program, National Center for Biotechnology Information (NCBI), National Library of Medicine, USA (http://www.ncbi.nlm.nih.gov/) (Altschul et al., 1997). Sequence alignments were performed by *Clustal W*1.83 XP (Thompson et al., 1997) and phylogenetic trees were constructed using neighbor-joining method (Saitou and Nei, 1987) using *MEGA6* software (Kumar et al., 2004).

### Sequence accession numbers

The 16S rRNA gene sequences obtained in this study have been deposited in GenBank under accession numbers AB793788–AB793797.

### Statistical analysis

Statistical Analysis was performed using the non-parametric Wilcoxon Rank-Sum test to compare traits shown as ranks in different isolates. Each trait was tested in triplicates. The statistical package used was Minitab version 17.

## Results

### Enumeration and isolation of rhizobacteria

Generally, the population density in this study was considerably higher in rhizoplane than in the rhizosphere for the 11 plant species. The population density of the total bacteria inhabiting the rhizoplane and the rhizosphere ranged from 1.1 × 10^3^ to 7.3 × 10^5^ CFUg^−1^ roots respectively representing a population of 30.11 and 69.89% respectively. The population density of spore formers inhabiting rhizoplane and rhizosphere ranged from 2 × 10^1^ to 1.3 × 10^4^ CFUg^−1^ roots respectively.

### *In vitro* evaluation of rhizospheric bacteria as plant growth promoters

Although many studies have been conducted to identify specific traits by which PGPR promote plant growth, usually they were limited to studying just one or two of these traits. Of the 531 total bacterial isolates of the present study only 66 were selected based on their ability to inhibit the world wide distributed soil-borne phytopathogenic fungi *F. oxysporum*, and *S. sclerotiorum*. All 66 selected isolates were further screened *in vitro* for a wide array of PGP traits. Tested PGP traits included N2-fixation, mineral phosphate and zinc solubilization, and IAA production.

**Figure 1:**
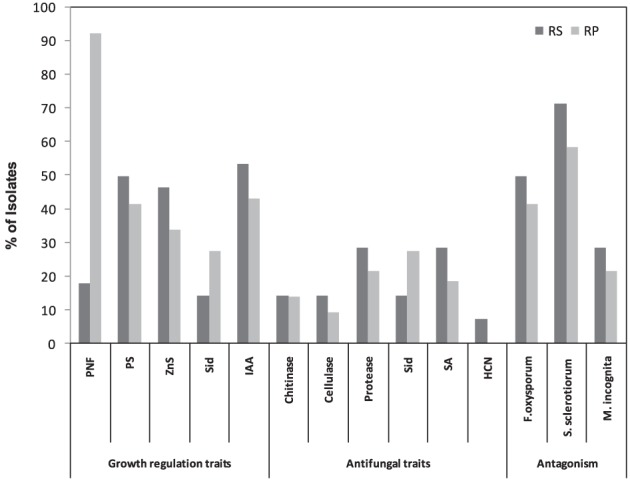
**Numerical data of the *in vitro* screening of bacterial isolates for plant growth promoting traits from rhizosphere (RS) and rhizoplane (RP) of different wild plant species**.

Among all isolates, 69.90% were found to be presumptive nitrogen fixers (PNFs). The percentages of PNFs among rhizosphere and rhizoplane isolates were 17.86%, and 92.3%, respectively (Figure 1). We tested the ability of the screened isolates to solubilize mineral phosphate (P) and zinc (Zn). As shown in Figure 1, 44.09 and 37.63% of the isolates were able to solubilize mineral P and Zn, respectively. These isolates varied in their solubilization abilities as being indicated by differences in solubilization index. On the other hand, proportions of rhizosphere and rhizoplane isolates that were able to solubilize P and Zn were 46.43 and 33.85%, respectively. In the present work, isolates varied greatly in their ability to produce IAA, with rhizosphere IAA producers being higher (53.57%) compared to rhizoplane ones (43.08%) (Figure 1).

### Antagonism toward plant pathogenic fungi and nematodes

PGPR have attracted the attention of many researchers because of the potential for developing these bacteria as inocula for plant disease control. Results presented in Figure 1 show that 44.09% of tested isolates had a wide range of antagonistic activity against *F. oxysporum*, while about 62.37% exhibited an antagonistic activity against *S. sclerotiorum*. Rhizosphere isolates were found to be highly efficient against fungal pathogens compared to rhizoplane ones. Of the rhizosphere isolates 50% were active against *F. oxysporum* and 71.43% against *S*. *sclerotiorum*, compared to 41.54% and 58.46% of the rhizoplane isolates respectively.

Cell free culture filtrates of isolated strains were also tested *in vitro* for their nematicidal activity on *M. incognita*. In general, juvenile mortality increased with increased exposure period to PGPR culture filtrates. A maximum nematode mortality of between 81% and 100% was observed. For reference, nematicidal Pseudomonads showed effects ranging from 84–96% to those of PGPR isolates. About 23.66% of the isolates showed nematicidal activity (Figure 1), with one isolate from rhizosphere and 4 isolates from rhizoplane exhibiting a significant reduction in the number of eggs hatching and a significant increase in *M. incognita* J2 mortality (97–100%). Interestingly, microscopic examination of the unhatched eggs indicated that a large proportion of them were severely damaged. However, this phenomenon was not observed in unhatched eggs treated with the reference strains.

### Screening of rhizospheric bacteria for antifungal and nematicidal traits

Concerning siderophores (Sid), 23.66% of the isolates were able to produce Sid. The percentage of Sid -producers among rhizoplane and rhizosphere isolates was 27.69% and 14.29% respectively (Figure 1). Results in Figure 1 also indicated the widespread ability of these isolates to produce SA (21.51%) (Figure 1). The results revealed that the proportion of HCN-producers varied among the isolates of different plant species and rhizosphere microhabitats. Only 6.45% of isolates produced HCN in rhizosphere habitats (Figure 1). Regarding the lytic enzymes, as shown in Figure 1, the number of protease- producers was the highest (23.66%) followed by chitinase- producers (13.89%), whereas cellulase-producers recorded the lowest number (10.75%) (Figure 1).

### Assessment of the *in vitro* PGPR traits

**Figure 2:**
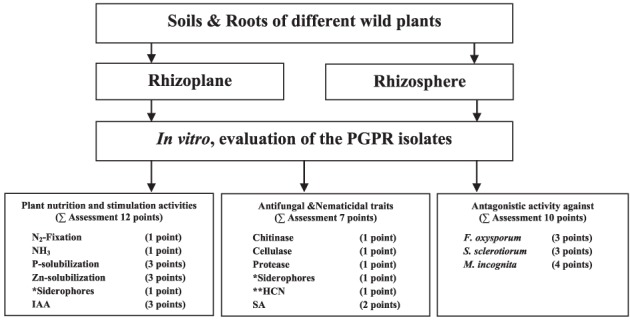
**A bonitur scale (of 29 points) used for the assessment of the isolates based on their *in vitro* PGP traits screening.** Two points were given to siderophores production, one as antifungal traits and one for facilitating iron uptake by plants. Points given to HCN production, if positive, were excluded from Σ assessment because it is considered ambiguous with the antifungal and nematicidal traits of HCN offset by deleterious effects on plant growth.

In an attempt to better select bacterial isolates with high plant growth promotion potential, a bonitur scale similar to that described by Krechel et al. (2002) was generated and used for the assessment of PGPR traits. In this scale, points are given for each *in vitro* bacterial trait examined. For traits examined here, the maximum possible bonitur score is 29 points (Figure 2). Results of the assessment revealed that out of the 66 isolates screened, 10 isolates according to Σ assessment values varied between 17 points and 23 points. Among those 10 isolates, 3 were isolated from rhizosphere, and 7 from rhizoplane of wild plants (Table 1). Isolate CSP03 from the wild plant *C. spinosa* showed the highest Σ assessment value of 23 points.

**Table 1:**
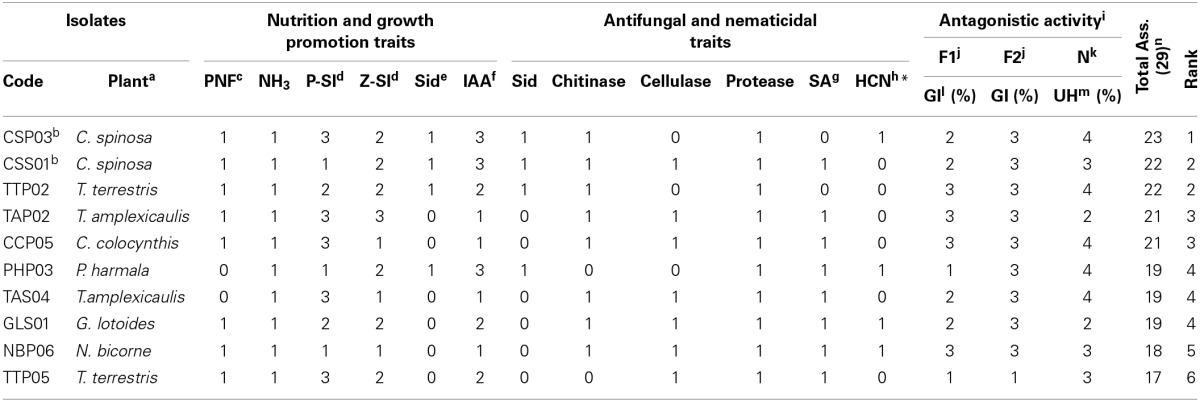
**Top 10 rhizosphere and rhizoplane isolates and their plant nutrition and growth promotion, antifungal and nematicidal traits, in addition to their antagonistic activity and general assessment and ranking for their ability to function as PGPR**.

^a^Wild Plant.

^b^S, Rhizosphere - P, Rhizoplane.

^c^PNF, Putative N_2_-fixation.

^d^SI, Phosphate solubilization index (1 = 2.99; 2 = 3 -4.99; 3 = 5), Zinc solubilization index (1 = 1.99; 2 = 2–3; 3 = 3).

^e^Sid, Siderophores production.

^f^IAA, Indoleacetic acid production (1 = 1.99; 2 = 2 −2.99; 3 = 3 μg ml^−1^).

^g^SA, Salicylic acid production (1 = 50–100; 2 = 100–200 μg ml^−1^).

^h^HCN, Hydrogen cyanide.

^i^Antagonistic activity against fungi & nematode.

^j^F_1_, Fusarium oxysporum, F_2_, Sclerotinia sclerotiorum.

^k^N, Meloidogyne incognita.

^l^GI (%), Growth inhibition percentage, (1 = 30–49.9%; 2 = 50–69.9%; 3 = 70%).

^m^UH (%), Unhatched % (1 = 30–49.9%; 2 = 50–69.9%; 3 = 70–89.9%; 4 = 90%).

^n^Total assessment points.

^*^The point given for HCN production was excluded.

### Molecular characterization of potential PGPR isolates in restriction analysis of the amplified 16S rRNA genes

**Figure 3:**
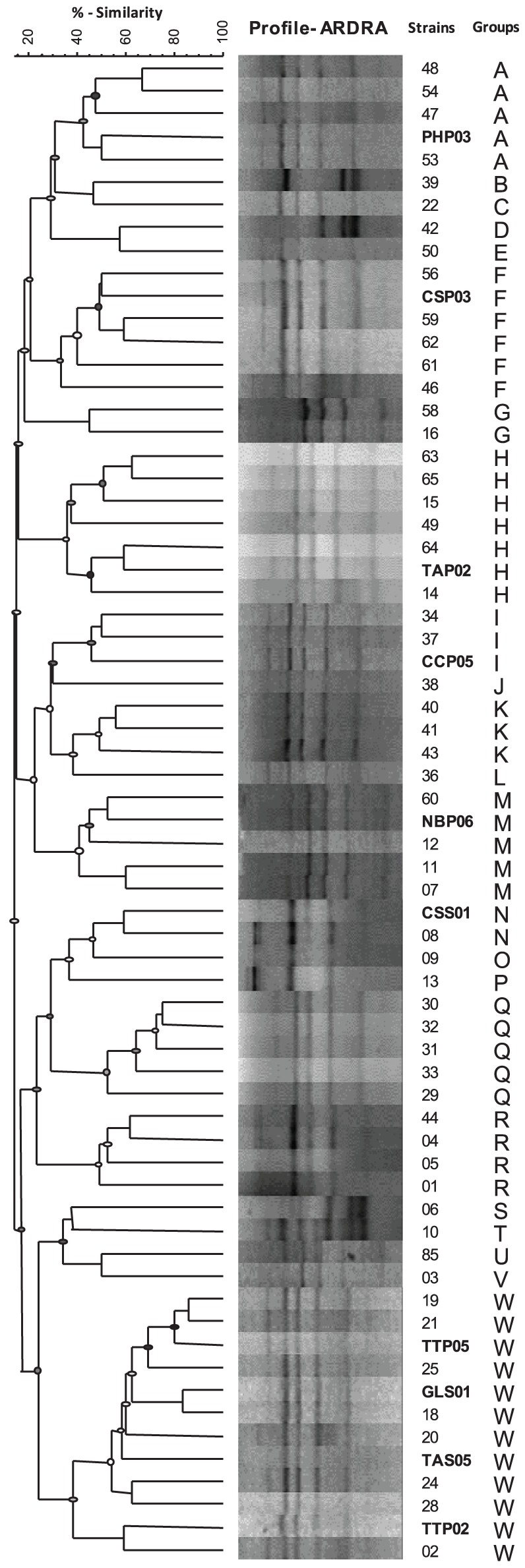
**Dendrogram of genetic similarity using Dice similarity coefficient index for bacterial isolates from rhizosphere and rhizoplane of different wild plant species.** ARDRA banding patterns were obtained after restriction digestion of the amplified 16S rRNA with *Hae*III. Banding patterns were analyzed with GelCompar software and dendrogram was constructed after grouping using UPGMA.

All of the 66 isolates that have been tested for PGPR traits were subjected to ARDRA and therefore were sorted into distinguished groups. Different profiles were generated by restriction digestion with the enzymes *Hae*III indicating the presence of different genotypes. In accordance with the dendrogram of genetic similarity using Dice similarity coefficient index, all 66 isolates were grouped into 23 different groups (Figure 3). Selected genotypes with highest PGP rank were therefore categorized and subjected to molecular identification using 16S rRNA gene partial sequence analysis.

### Molecular typing and phylogenetic affiliation of potential PGPR isolates

Representative isolates belonging to different ARDRA groups, showing high growth promoting potential, were selected for the identification purposes. Isolates were identified by partial sequencing of their 16S rRNA genes, which led to the classification of the isolates into three specific genera, namely; *Bacillus*, *Enterobacter*, and *Pseudomonas* (Table 2).

**Table 2:** **Genotypic analysis and assignment of 16S rRNA gene sequence for selected isolates with *in vitro* PGPR traits**.

**ARDRA profile**	**No. of Strains^a^**	**Code^b^**	**Blast match**			
			**Identity**	**Accession no^c^**	**Similarity (%)**	**Accession no^d^**
F	6	CSP03	*Pseudomonas stutzeri* GAPP4	GU396288	98.11	AB793795
N	2	CSS01	Bacillus sp. ZB2	EU236757	99.58	AB793788
W	12	TTP02	*Bacillus subtilis* LLS-M3-11	HM744709	99.40	AB793794
H	7	TAP02	*Enterobacter* sp. LCR37	FJ976546	94.00	AB793792
I	3	CCP05	*Enterobacter cloacae* 1245	JF322972	95.53	AB793791
A	5	PHP03	*Pseudomonas putida* BBAL5-01	FJ217182	96.82	AB793789
W	12	TAS04	*Bacillus subtilis* subsp. *inaquosorum* M61	JF411298	99.20	AB793793
W	12	GLS01	*Bacillus subtilis* subsp. *inaquosorum* KTH-61	HM854250	99.60	AB793790
M	5	NBP06	*Enterobacter* sp. G8-6	HM217970	99.19	AB793797
W	12	TTP05	*Bacillus subtilis* CD-6	EU090295	97.95	AB793796

a Numbers of strains in the corresponding ARDRA profile.

b Code for the selected strains with best PGP traits.

c GeneBank sequence accession numbers of most closely related sequences.

d GeneBank sequence accession numbers of selected strains.

**Figure 4:**
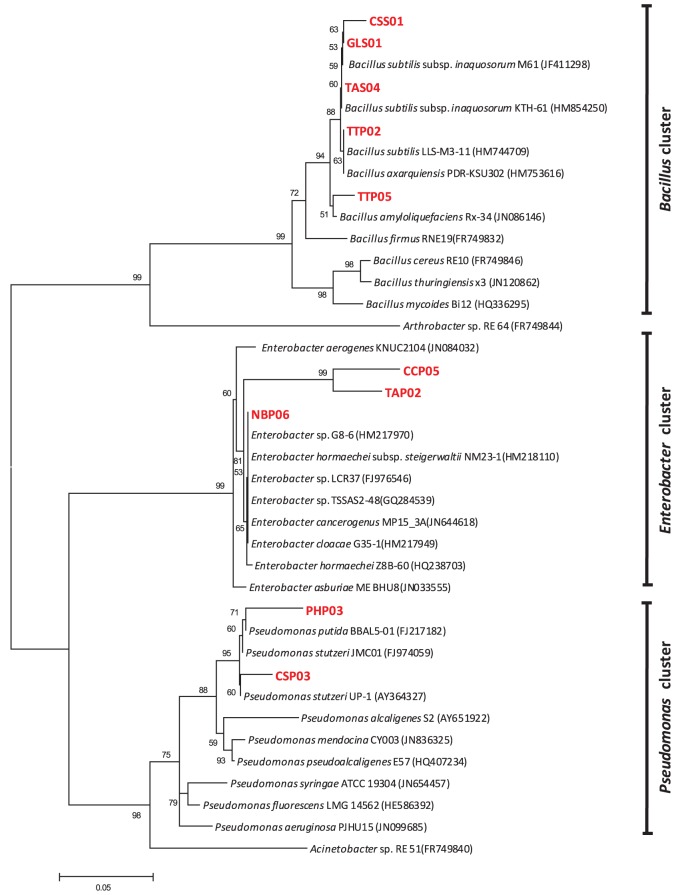
**Neighbor-joining tree showing the phylogenetic relationship between selected potential PGPR isolates and reference strains from GenBank database.** The bar represents 0.05 substitutions per site, bootstrap values (*n* = 1000) are displayed.

Phylogenetic studies confirmed the affiliation of the selected genotypes into *Bacillus*, *Enterobacter*, and *Pseudomonas* clusters revealed by clustering of each genus to its corresponding group. Figure 4 represents the phylogenetic tree based on 16S rRNA sequences analysis and showing the relationship between selected isolates and representative species along with other related genera. Although most of the genotypes were closely related to reference strains, three strains designated as PHP03, CCP05, and TAP02 were regarded as novel species based on their low similarity and high bootstrap values.

According to Blast similarity matches, the *Bacillus* group was found to include five different strains, CSS01 (99.58% similarity to *Bacillus* sp. strain ZB2), GLS01 (99.6% similarity to *Bacillus subtilis* strain KTH-61), TAS04 (99.2% similarity to *Bacillus subtilis* strain M61), TTP02 (99.4% similarity to *Bacillus subtilis* strain LLS- M3-11), and TTP05 (97.95% similarity to *Bacillus subtilis* strain CD-6). The five representative *Bacillus* strains showed a high growth promoting potentials. In this study we have demonstrated the dominance of endospore-forming bacilli in rhizospheric arid soil and confirmed their potential as PGPR. The tested antifungal traits also confirmed the high ranking of all *Bacillus* strains as a PGPR isolates. The distribution of *Bacillus* strains in rhizosphere was found to be much higher than in rhizoplane perhaps due to their higher tolerance to arid conditions and therefore independent survival from plant roots.

*Enterobacter* group on the other hand was found to include three different strains: CCP05 (95.53% similarity *to Enterobacter cloacae* strain 1245), TAP02 (94% similarity to *Enterobacter asburiae* strain E53), and NBP06 (99.1% similarity to *Enterobacter cloacae* strain G35-1). The three tested strains also possessed plant growth promoting and anti-phytopathogenic fungi traits. The third group included the Pseudomonades which had two different strains: PHP03 (96.82% similarity to *Pseudomonas stutzeri* strain p4) and CSP03 (98.11% similarity to *P. stutzeri* strain GAPP4). *Pseudomonas* strains PHP03 and CSP03 showed a high assessment rank for nutrition and growth regulation traits. The two strains were characterized for their higher nematicidal and fungicidal effects.

## Discussion

A considerable worldwide research has focused on the exploration of varied agro- ecological niches for the existence of native beneficial micro-organisms. (Lugtenberg and Kamilova, 2009; Dastager et al., 2010). Wild plants are likely to harbor unique rhizobacterial communities that differ from those found in cultivated plants, which are extensively bred and subjected to intensive applications of various agrochemicals (Gamal-Eldin et al., 2008).

In the current study, rhizobacteria with *in vitro* PGPR traits were isolated from arid rhizospheric soil of 11 different wild plant species. These potential PGPR isolates were enumerated and screened *in vitro* for a broad spectrum of plant growth-promoting abilities as well as for antagonistic potential against phytopathogenic fungi and nematodes. All isolates were grouped according to their ARDRA banding pattern. Selected strains scoring high rank as PGPR were identified and their phylogenetic affiliations were determined.

In general, the population density of both bacteria and spore formers isolated from the 11 plant species in the present study was higher in the rhizoplane than in the rhizosphere. Differences in numbers and composition of rhizosphere microbes for different plant species and even different varieties within species have been reported (Kremer et al., 1990; Bisseling et al., 2009). The higher numbers of bacteria in the rhizoplane of the arid soil may be attributed to the presence of the root exudates that favor their existence. Rhizosphere on the other hand was found to harbor higher spore formers bacteria compared to rhizoplane. This may be best explained on the ground of lacking root exudates that support microbial activity.

Isolation of bacteria from rhizosphere and rhizoplane usually results in a large number of strains. It is almost impossible to test and evaluate every bacterial strain isolated for all PGP traits. In this study, a strategy has been established to sort out bacterial strains and restrict them to those highly suspected to have PGP traits. Antagonistic potential against phytopathogenic fungi was selected as criteria to have a preliminary judgment for the isolates whether they possess PGP traits or not. Amongst 531 bacteria isolated from the rhizospheric soil, only 66 isolates were able to inhibit *F. oxysporum* and *S. sclerotiorum* and were therefore recruited as PGPR. Isolates having antagonist potentials were considered as good candidates for being PGPR because of their indirect effect for promoting plant growth via inhibition of various plant pathogens. Selected isolates were further screened for PGPR traits like N2-fixation, mineral phosphate and zinc solubilization, and IAA production. 69.90% of the isolates were found to be nitrogen fixers with 92.3% isolated from the rhizoplane. Biological N2- fixation (BNF) by soil microorganisms is considered one of the major mechanisms by which plants benefit from the association of micro-partners. Several PGPR have been reported to fix N2 (Andrade et al., 1997). Lugtenberg and Kamilova (2009) attributed a similar distribution of nitrogen fixers to chemo-attraction of the microorganisms moving toward carbon exudates, allowing them to colonize and multiply in both the rhizosphere and the rhizoplane. Malik et al. (1997) investigated the nitrogen fixing ability of *Azospirillum* strain N-4 in rice and found a significant contribution of nitrogen fixed by the PGPR.

In soil, both macro and micronutrients undergo a complex dynamic equilibrium between soluble and insoluble forms. The equilibrium is strongly influenced by the soil pH which can be shifted by the microbiota ultimately affecting their accessibility to plant roots for absorption. Soluble phosphorus and zinc are deficient in most natural soils, However, insoluble forms of phosphorus and zinc can be made available to plants by the aids of rhizospheric microorganisms (Saravanan et al., 2003).

In the case of P and Zn solubilization, 44.09 and 37.63% of the isolates from the present study had such traits respectively. Proposed mechanisms for solubilization abilities have been reported, among which production of organic acids was the major mechanism of action by which insoluble compounds were converted to more soluble forms (Pietr et al., 1990).

IAA is also one of the most physiologically active growth regulator (auxins), and a common product of L-tryptophane that is metabolized by several microorganisms including PGPR (Arshad and Frankenberger, 1991). The present study showed that the percentage of rhizosphere IAA producers was higher compared to that of the rhizoplane. Several studies proved a good correlation between induction of root elongation and phytohormone production (Asghar et al., 2002). It is hypothesized that the rhizospheric bacteria influenced the growth of wild plants by producing auxins in the plant rhizotic zone upon the release of tryptophan in the root exudates (Asghar et al., 2002).

Besides stimulating plant growth by direct mechanisms, PGPR can also indirectly induce plant growth by protecting plants against soil-borne pathogens (Bloemberg and Lugtenberg, 2001; Mercado-Blanco and Bakker, 2007). Rhizosphere isolates showed higher antagonistic activity against *F. oxysporum* and *S. sclerotiorum* compared to those isolated from the rhizoplane. It has been reported previously that the plant species or cultivars and especially the composition of root exudates plays a key role in the diversity of rhizobacterial populations and can influence the frequency of antagonistic bacteria (Kremer et al., 1990; Siciliano et al., 1998).

Nematicidal activity against *M. incognita* was also observed especially by the rhizosphere isolates. Similar results were reported by Bin et al. (2005) who found that whole cultures and culture filtrates of some rhizobacteria showed nematicidal effects on J2 of *M. javanica* ranging from 62–64 and 62–70% respectively. This finding provides evidence that such isolates may produce nematicidal compounds to control *M. incognita.* The damage shown under the microscope by unhatched eggs was presumably due to lytic enzymes secreted by the tested isolates. In this context, Khan et al. (2004) found that *M. javanica* eggs treated with chitinase or protease in a liquid culture of *Paecilomyces lilacinus* displayed large vacuoles in the chitin layer. This phenomenon may also be the case in the top 5 isolates in our studies as they were positive for chitinase and protease production.

PGPR may enhance plant growth *via* suppression of phytopathogens by a variety of indirect mechanisms. This may include, the ability to produce siderophores to chelate iron, antifungal metabolites, lytic enzymes (e.g., chitinase, protease, cellulase and β-1, 3- glucanase), and hydrogen cyanide (Bloemberg and Lugtenberg, 2001). Among the total PGPR isolates, 23.66% were Sid-producers with higher percentage being rhizoplane isolates. It has been reported that the ability of PGPR to antagonize pathogenic fungi was related to the production of extracellular siderophores which deprive phytopathogenic microflora of iron, thus limiting their growth (Kloepper et al., 1980).

Many studies indicated that SA also plays an important role in plant defense response against pathogen attack and is essential for the development of both systemic acquired resistance (SAR) and induced systemic resistance (ISR) in plants (Zhang et al., 2002; Elbadry et al., 2006). A percentage of 21.51% of our isolates showed ability to produce SA.

Cyanide production is an ambiguous trait and is sometimes associated with deleterious as well as beneficial rhizobacteria (Bakker and Schippers, 1987; Alström and Burns, 1989). Only 6.45% of our rhizosphere isolates were HCN-producers.

Lytic enzymes is another trait associated with PGPR enabling them to limit fungal pathogens growth, as *in vitro* studies showed that the exposure of selected plant pathogenic fungi to lytic enzymes such as chitinase, protease, gluconase or cellulase can result in degradation of the structural matrix of fungal cell wall (Dunne et al., 1998). In our study, we observed that 23.66% were protease-producers, 13.89% were chitinase- producers and only 10.75% were cellulose-producers.

During the molecular study, 66 isolates were genotyped using amplified rDNA restriction analysis (ARDRA and 16S rRNA gene sequence analysis. The taxonomic composition of the representative genotypes from both rhizosphere and rhizoplane comprised *Bacillus*, *Enterobacter*, and *Pseudomonas*.

*Bacillus* spp. is known for their wide distribution in many soil types and was suggested for their possible role in the adaptation of desert plants to support their growth (Jorquera et al., 2012). Among all *Bacillus* spp. that have been isolated and characterized as PGPR, spore-forming bacilli have received much attention for commercial purposes due to their stability in the environment (Brannen and Backman, 1993).

Mirza et al. (2001) reported the isolation of strains of *Enterobacter* spp. from rhizosphere of sugarcane and illustrated their ability to function as PGPR. *Enterobacter* spp. were also found in diversity of PGPR isolated from sugarcane cultivated in South of Brazil (Beneduzi et al., 2013). In an effort to study the diversity of PGPR associated with rhizopheric soil and roots of canola, Farina et al. (2012) found that *Pseudomonas* and *Enterobacter* were among the most abundant rhizospheric bacteria showing several PGPR traits.

It has also been reported that *Pseudomonas* spp. is one of the most important bacteria inhabiting the rhizosphere of a diverse group of plants (Costa et al., 2006).

It has been shown that, in comparison to other plant microenvironments, the rhizosphere is one of the main reservoirs of antagonistic bacteria (Berg et al., 2005) with Pseudomonads as being most dominant bacteria showing antagonistic properties and therefore potential PGPR (Berg et al., 2006). However, in contrast to these reports, our results suggest that rhizoplane of wild plants in arid soil is a potential source for Pseudomonades with PGP properties rather than rhizosphere. *Pseudomonas aeruginosa* was found to play important role in oxidative stress tolerance in wheat (Islam et al., 2014). Moreover, Jorquera et al. (2012) found more diverse flora of rhizobacterial composition of the ancient clones of *L. tridentata*, with the hypothesis that those might play an important role in the adaptation of this plant and others to arid environments.

## Conclusion

Rhizobacteria isolated from native wild desert plants was found to harbor antagonistic activity against *F*. *oxysporum* and *S*. *sclerotiorum* as well as unique PGP traits e.g., N2-fixation, indoleacetic acid and siderophores production, mineral phosphate and zinc solubilization, and antagonistic potentials. These antagonistic strains with their broad spectrum of *in vitro* PGP abilities and antagonistic potentials in such harsh environment make them good candidates as growth supporting agents for plants thriving at desert environment. As, *in vitro* studies should be considered prior to any green house and field studies, the present study provides a compelling evidence that rhizobacteria associated with plants growing at harsh environments do possess the traits which may contribute to plant adaptation to arid environments. Characterizing strains with *in vitro* PGPR traits from native plants adapted to harsh environment like that of Saudi Arabia would help scientists understand the behavior of PGPR in extreme environments and also funding strains that can be used in such environment to improve the growth of agricultural plants.

### Conflict of interest statement

The authors declare that the research was conducted in the absence of any commercial or financial relationships that could be construed as a potential conflict of interest.
